# Trends in Esophageal Cancer Mortality and Stage at Diagnosis by Race and Ethnicity in the United States

**DOI:** 10.1007/s10552-021-01443-z

**Published:** 2021-05-18

**Authors:** Edgar Corona, Liu Yang, Eric Esrailian, Kevin A. Ghassemi, Jeffrey L. Conklin, Folasade P. May

**Affiliations:** 1 Department of Medicine, University of California, San Francisco, CA USA; 2 The Vatche and Tamar Manoukian Division of Digestive Diseases, Department of Medicine, UCLA Robert G. Kardashian Center for Esophageal Health, David Geffen School of Medicine, University of California, Los Angeles, CA USA; 3 Division of Gastroenterology, Department of Medicine, VA Greater Los Angeles Healthcare System, Los Angeles, CA USA; 4 Jonsson Comprehensive Cancer Center, UCLA Kaiser Permanente Center for Health Equity, Cancer Prevention Control Research, UCLA, Los Angeles, CA USA

**Keywords:** Esophageal cancer, Health disparities, Mortality, Stage at diagnosis

## Abstract

**Introduction:**

Esophageal cancer (EC) is an aggressive malignancy with poor prognosis. Mortality and disease stage at diagnosis are important indicators of improvements in cancer prevention and control. We examined United States trends in esophageal adenocarcinoma (EAC) and esophageal squamous cell carcinoma (ESCC) mortality and stage at diagnosis by race and ethnicity.

**Methods:**

We used Surveillance, Epidemiology, and End Results (SEER) data to identify individuals with histologically confirmed EAC and ESCC between 1 January 1992 and 31 December 2016. For both EAC and ESCC, we calculated age-adjusted mortality and the proportion presenting at each stage by race/ethnicity, sex, and year. We then calculated the annual percent change (APC) in each indicator by race/ethnicity and examined changes over time.

**Results:**

The study included 19,257 EAC cases and 15,162 ESCC cases. EAC mortality increased significantly overall and in non-Hispanic Whites from 1993 to 2012 and from 1993 to 2010, respectively. EAC mortality continued to rise among non-Hispanic Blacks (NHB) (APC = 1.60, *p* = 0.01). NHB experienced the fastest decline in ESCC mortality (APC = − 4.53, *p* < 0.001) yet maintained the highest mortality at the end of the study period. Proportions of late stage disease increased overall by 18.5 and 24.5 percentage points for EAC and ESCC respectively; trends varied by race/ethnicity.

**Conclusion:**

We found notable differences in trends in EAC and ESCC mortality and stage at diagnosis by race/ethnicity. Stage migration resulting from improvements in diagnosis and treatment may partially explain recent trends in disease stage at diagnosis. Future efforts should identify factors driving current esophageal cancer disparities.

**Supplementary Information:**

The online version contains supplementary material available at 10.1007/s10552-021-01443-z.

## Introduction

Esophageal cancer (EC) is an aggressive and deadly malignancy [1]. In 2020, an estimated 18,440 Americans were diagnosed with and 16,170 died from the disease [2]. The two major EC histological subtypes, esophageal squamous cell carcinoma (ESCC) and esophageal adenocarcinoma (EAC), have distinct epidemiology and risk factors [3, 4]. Historically, ESCC has had higher incidence and mortality than EAC in the U.S.; however, EAC incidence now exceeds ESCC incidence largely due to increasing prevalence of EAC risk factors, including obesity, gastroesophageal reflux disease (GERD), and Barrett metaplasia [3, 4]. While tobacco use is a risk factor for both EAC and ESCC, it is more strongly associated with ESCC [3, 4]. Thus, a decreasing prevalence of heavy tobacco use nationally also likely contributes to the decline in ESCC incidence [5]. Currently, 70% of EC cases in the U.S. are EAC [6].

The literature describes existing racial and ethnic (racial/ethnic) disparities in EC incidence by histological subtype [6–8]. From 2010 to 2014, EAC incidence was 2.6, 5, and 5.9 times higher in White males than in Hispanic, Black, and Asian males, respectively [6]. Over this same time period, EAC incidence decreased significantly in males overall yet remained stable among White males [6]. ESCC incidence is 3–4 times higher in Black males than White males, and Black males have higher ESCC incidence than males in other racial/ethnic groups despite experiencing the fastest decline in incidence over time [6, 7]. Although both ESCC and EAC incidence are higher in men than women, the relative incidence by race/ethnicity among women is similar to the pattern observed for men [6].

Like incidence, mortality and disease stage at diagnosis are two important cancer control indicators. Both provide insight into national progress toward maximizing early detection, optimizing treatment, and reducing health disparities. However, racial/ethnic differences in EAC and ESCC mortality and disease stage at diagnosis are currently understudied. In this study, we evaluate recent U.S. trends in EAC and ESCC mortality and stage at diagnosis by race/ethnicity. Findings will help determine whether advances in technology and treatment have improved EAC and ESCC outcomes equitably and direct future efforts to reduce racial/ethnic disparities.

## Methods

### Data source and study population

We used National Cancer Institute (NCI) Surveillance, Epidemiology, and End Results (SEER) 13 Program data, which includes tumor registry data from 13 U.S. regions from 1992 to 2016 and represents 13.4% of the U.S. population [9]. In 1992, SEER broadened its geographic and demographic coverage to increase representation of racial/ethnic minority groups, documenting Hispanic ethnicity and more detailed racial designations (White, Black, Asian/Pacific Islander, and American Indian/Alaska Native), foreign-born, and urban populations [10].

### Cancer indicators

Our primary outcomes were all-cause mortality and stage at diagnosis for EAC and ESCC. For all-cause mortality, we used the incidence-based mortality SEER 13 registry [11] (1993–2016) to find adult cases meeting our inclusion criteria: (1) histologically confirmed diagnosis, (2) age ≥ 20 at time of death, and (3) International Classification of Diseases for Oncology, Third edition (ICD-O-3) code diagnosis of EAC (codes 8140–8141, 8143, 8145, 8147, 8480, 8481, 8560, 8562, 8570–8575) or ESCC (codes 8070–8078) [12]. We excluded cases reported only through autopsy or death certificates [12].

For stage at diagnosis, we used the incidence SEER 13 registry [13] (1992–2015) to identify cases meeting the following inclusion criteria: (1) histologically confirmed diagnosis, (2) age ≥ 20 at time of diagnosis, and (3) International Classification of Diseases for Oncology, Third edition (ICD-O-3) code diagnosis of EAC (codes 8140–8141, 8143, 8145, 8147, 8480, 8481, 8560, 8562, 8570–8575) or ESCC (codes 8070–8078). We used the SEER Historic stage A variable to estimate the proportion of cases that presented at each disease stage as three categories: unstaged, early stage (localized), and late stage (regional or distant) [14]. We reasoned that comparing unstaged, local, and late stage disease was most clinically interesting given that treatment guidelines recommend surgical resection with curative intent for T1-2N0 (local) disease, while the optimal management for disease stages > T2N0 (regional and distant) is less defined [15].

Consistent with prior studies, we did not include death cases occurring in 1992 for mortality analyses so that the mortality cohort more closely matched the cohort used for stage at diagnosis analyses [16, 17]. Thus, we report trends in mortality from 1993 to 2016 and trends in stage at diagnosis from 1992 to 2015. For race/ethnicity, we used five mutually exclusive categories: Hispanics, non-Hispanic Whites (NHW), non-Hispanic Blacks (NHB), non-Hispanic Asian/Pacific Islanders (NHAPI), and non-Hispanic American Indians/Alaskan Natives (NHAI/AN).

### Statistical analysis

We used SEER*Stat version 8.3.5 to calculate annual and overall EAC and ESCC mortality rates (per 100,000 persons), which were age-standardized to the 2,000 U.S. population. We also obtained mortality rate ratios (RR), a measure of relative mortality that compares each racial/ethnic group to NHW [18]. We estimated 95% confidence intervals (CIs) for RRs using the methods outlined in Tiwari et al. and performed tests of significance in SEER*Stat [19]. Using SAS version 9.4, we conducted a chi-square test to compare the proportion of late stage and unstaged disease among all racial/ethnic groups and then performed pairwise chi-square comparisons between NHW and each other racial/ethnic group.

Following these analyses, we used the NCI’s Joinpoint Program Version 4.6.0.0 to examine temporal trends for mortality and stage at diagnosis by race/ethnicity and histological subtype. We performed joinpoint regression, which fits a series of joined least-squares regression lines to the natural logarithm of the rates/proportions plotted against time, to identify changes in trends. The Joinpoint Program uses a sequence of permutation tests to determine the best number of joinpoints with up to four joinpoints allowed [20]. The program also calculates the annual percent change (APC) in each fitted segment to quantify changes in rates/proportions [21]. An APC that is significantly different from zero indicates a statistically significant change in rates/proportions over the specific time period. Finally, we performed parallel pairwise comparison tests to examine whether the average annual percent change (AAPC) of EAC and ESCC mortality over the entire time period was significantly different for each racial/ethnic group compared to non-Hispanic Whites [22]. All significance tests were two-sided, and *p* values less than 0.05 were considered statistically significant.

## Results

### EAC mortality and stage at diagnosis by race/ethnicity and sex

There were 16,562 EAC cases included in the mortality analysis and 19,257 EAC cases included in the stage at diagnosis analysis (Table 1). EAC mortality was highest in NHW and lowest in NHAPI in males and females combined [NHW vs. NHAPI: 3.2 (95% CI) (3.1–3.2) vs. 0.6 (0.5–0.6) deaths per 100,000] (Table 2). Among males only, mortality was highest in NHW and lowest in NHAPI [NHW vs. NHAPI: 6.2 (6.1–6.3) vs 1.0 (0.9–1.2) deaths per 100,000]. However, in females only, EAC mortality was highest in both NHW and NHAI/AN and lowest in NHAPI [NHW vs. NHAI/AN vs. NHAPI: 0.8 (0.8–0.8) vs. 0.8 (0.5–1.3) vs. 0.2 (0.2–0.2) deaths per 100,000]. EAC mortality was significantly lower in all racial/ethnic groups than in NHW in males and females combined (Table 2).

**Table 1:** Esophageal adenocarcinoma and squamous cell carcinoma cases by race/ethnicity

	1993–2016 Mortality analytic sample count (%)	1992–2015 Stage at diagnosis analytic sample count (%)
EAC		
NHW	14,503 (87.6)	16,853 (87.5)
Hispanic	1,100 (6.6)	1,285 (6.7)
NHB	441 (2.7)	493 (2.6)
NHAPI	414 (2.5)	484 (2.5)
NHAI/AN	93 (0.6)	111 (0.6)
Unknown	11 (0.1)	31 (0.2)
Total	16,562 (100.0)	19,257 (100.0)
ESCC		
NHW	7,629 (55.3)	8,431 (55.6)
Hispanic	1,010 (7.3)	1,133 (7.5)
NHB	3,416 (24.8)	3,646 (24.0)
NHAPI	1,594 (11.6)	1,795 (11.8)
NHAI/AN	126 (0.9)	139 (0.9)
Unknown	13 (0.1)	18 (0.1)
Total	13,788 (100.0)	15,162 (100.0)

*EAC* Esophageal Adenocarcinoma, *ESCC* Esophageal Squamous Cell Carcinoma, *NHW* Non-Hispanic White, *NHB* Non-Hispanic Black, *NHAPI* Non-Hispanic Asian and Pacific Islander, *NHAI/AN* Non-Hispanic American Indian/Alaska Native

**Table 2:** Esophageal adenocarcinoma and squamous cell carcinoma mortality rates and proportions of late stage and unstaged disease at diagnosis in the United States by sex and race/ethnicity

	Incidence-based mortality per 100,000 (95% CI), 1993–2016				Late stage proportion (%) at diagnosis (95% CI), 1992–2015				Unstaged proportion (%) at diagnosis (95% CI), 1992–2015			
	IBM Both sexes	Rate ratio^a^	IBM Male	IBM Female	Both sexes	*p* value*	Male	Female	Both sexes	*p* value*	Male	Female
EAC												
OVERALL	2.5 (2.5–2.5)	0.8 (0.8–0.8)	4.9 (4.8–5.0)	0.7 (0.6–0.7)	65.5 (64.8–66.1)	0.801	66.5 (65.7–67.2)	59.8 (57.9–61.6)	10.8 (10.4–11.3)	0.208	10.0 (9.5–10.4)	15.5 (14.2–16.9)
NHW	3.2 (3.1–3.2)	REF	6.2 (6.1–6.3)	0.8 (0.8–0.8)	65.3 (64.6–66.1)	REF	66.4 (65.6–67.2)	59.1 (57.2–61.1)	10.4 (10.0–10.9)	REF	9.6 (9.1–10.1)	15.1 (13.6–16.5)
HISPANIC	1.5 (1.4–1.6)	0.5 (0.5–0.5)	3.1 (2.9–3.3)	0.4 (0.3–0.5)	65.3 (60.9–69.5)	0.992	67.7 (62.6–72.4)	58.4 (49.3–67.2)	13.6 (11.7–15.5)	< 0.001	13.0 (11.0–15.0)	17.4 (11.9–22.9)
NHB	0.8 (0.7–0.9)	0.2 (0.2–0.3)	1.4 (1.2–1.5)	0.3 (0.3–0.4)	66.6 (64.0–69.2)	0.353	67.2 (64.4–70.0)	63.0 (55.6–70.0)	15.6 (12.4–18.8)	< 0.001	12.5 (9.1–15.9)	24.8 (17.2–32.4)
NHAPI	0.6 (0.5–0.6)	0.2 (0.2–0.2)	1.0 (0.9–1.2)	0.2 (0.2–0.2)	69.2 (64.9–73.3)	0.077	69.8 (65.0–74.3)	66.7 (55.8–76.4)	12.6 (9.7–15.6)	0.120	11.6 (8.4–14.7)	17.2 (9.3–25.2)
NHAI/AN	1.8 (1.4–2.2)	0.6 (0.4–0.7)	3.0 (2.3–3.8)	0.8 (0.5–1.3)	66.7 (57.1–75.3)	0.769	63.1 (51.9–73.4)	77.8 (57.8–91.4)	5.4 (1.2–9.6)	0.085	7.1 (1.6–12.7)	0.0
ESCC												
OVERALL	2.1 (2.1–2.1)	1.2 (1.2–1.3)	3.1 (3.0–3.1)	1.3 (1.3–1.4)	60.1 (59.3–60.9)	0.012	62.8 (61.9–63.8)	55.2 (53.9–56.5)	16.1 (15.5–16.7)	0.485	14.6 (13.9–15.3)	18.6 (17.6–19.6)
NHW	1.7 (1.6–1.7)	REF	2.2 (2.2–2.3)	1.2 (1.2–1.3)	58.4 (57.3–59.4)	REF	62.2 (60.8–63.5)	53.2 (51.6–54.9)	16.4 (15.6–17.2)	REF	14.5 (13.5–15.4)	19.1 (17.8–20.4)
HISPANIC	1.5 (1.4–1.6)	0.9 (0.8–0.9)	2.6 (2.4–2.8)	0.6 (0.6–0.7)	59.8 (56.9–62.6)	0.378	61.5 (58.2–64.8)	54.6 (48.8–60.4)	19.2 (16.9–21.4)	0.021	18.6 (16.0–21.2)	20.8 (16.1–25.5)
NHB	5.9 (5.7–6.1)	3.5 (3.4–3.7)	9.7 (9.3–10.1)	3.3 (3.1–3.5)	61.5 (59.9–63.1)	0.001	62.7 (60.8–64.6)	58.9 (56.1–61.8)	15.2 (14.1–16.4)	0.101	14.4 (13.0–15.8)	17.0 (14.8–19.1)
NHAPI	2.2 (2.1–2.3)	1.3 (1.2–1.4)	3.9 (3.7–4.1)	0.9 (0.8–0.9)	64.8 (62.5–67.0)	< 0.001	66.1 (63.6–68.6)	60.7 (56.1–65.3)	14.3 (12.7–15.9)	0.028	13.1 (11.3–14.9)	18.4 (14.7–22.0)
NHAI/AN	2.5 (2.1–3.0)	1.5 (1.2–1.8)	4.3 (3.4–5.3)	1.2 (0.8–1.7)	70.5 (62.2–77.9)	0.004	67.3 (58.1–76.6)	78.0 (65.4–90.7)	8.6 (4.0–13.3)	0.014	11.2 (5.0–17.5)	2.4 (0.0–7.2)

Rates are age-adjusted to the 2000 U.S. standard population (19 age groups-census p25-1130)

*95% CI* 95% confidence interval, *REF* Reference Group, *EAC* Esophageal Adenocarcinoma, *ESCC* Esophageal Squamous Cell Carcinoma, *IBM* Incidence-based Mortality, *OVERALL* Total data of all racial/ethnic groups combined, *NHW* Non-Hispanic White, *NHB* Non-Hispanic Black, *NHAPI* Non-Hispanic Asian and Pacific Islander, *NHAI/AN* Non-Hispanic American Indian/Alaska Native

*p values of pairwise chi-square comparisons of proportions of disease stage in both sexes by race/ethnicity compared to the reference group, non-Hispanic White

^a^Rate ratios and 95% confidence intervals of incidence-based mortality rates in both sexes by race/ethnicity compared to the reference group, non-Hispanic White

Overall, 65.5% [64.8–66.1%] of individuals with EAC were diagnosed with late stage disease, and there were no significant differences by race/ethnicity (Table 2). 10.8% [10.4–11.3%] of individuals with EAC had unstaged disease with significant differences by race/ethnicity. Hispanics [13.6% (11.7–15.5%)] and NHB [15.6% (12.4–18.8%)] had significantly larger proportions of unstaged disease at diagnosis than NHW [10.4% (10.0–10.9%)] (Table 2).

### ESCC mortality and stage at diagnosis by race/ethnicity and sex

There were 13,788 ESCC cases in the mortality analysis and 15,162 ESCC cases in the stage at diagnosis analysis (Table 1). ESCC mortality per 100,000 persons was significantly higher in NHB [5.9 (5.7–6.1)], NHAPI, [2.2 (2.1–2.3)], and NHAI/AN [2.5 (2.1–3.0)] but significantly lower for Hispanics [1.5 (1.4–1.6)] compared to NHW [1.7 (1.6–1.7)]. Notably, ESCC mortality was over three times higher in NHB than NHW [RR = 3.5, (3.4–3.7)] (Table 2); the RR reached 4.3 [4.1–4.5] when comparing NHB males to NHW males (data not shown). Hispanics had the lowest ESCC mortality among all racial/ethnic groups [1.5 (1.4–1.6) deaths per 100,000].

Among ESCC cases, 60.1% (59.3–60.9%) presented with late stage disease (Table 2). ESCC stage at diagnosis was significantly different by race/ethnicity: NHB [61.5%, (59.9–63.1%)], NHAPI [64.8% (62.5–67.0%)], and NHAI/AN [70.5% (62.2–77.9%)] had significantly greater proportions of late stage disease at diagnosis than NHW [58.4% (57.3–59.4%)]. ESCC cases were unstaged in 16.1% (15.5–16.7%) of individuals. Hispanics [19.2% (16.9–21.4%)] had a significantly greater proportion of unstaged ESCC compared to NHW [16.4% (15.6–17.2%)], while NHAPI [14.3% (12.7–15.9%)] and NHAI/AN [8.6% (4.0–13.3%)] had significantly lower proportions of unstaged ESCC (Table 2).

### Trends in EAC mortality overall and by race/ethnicity

**Fig. 1:**
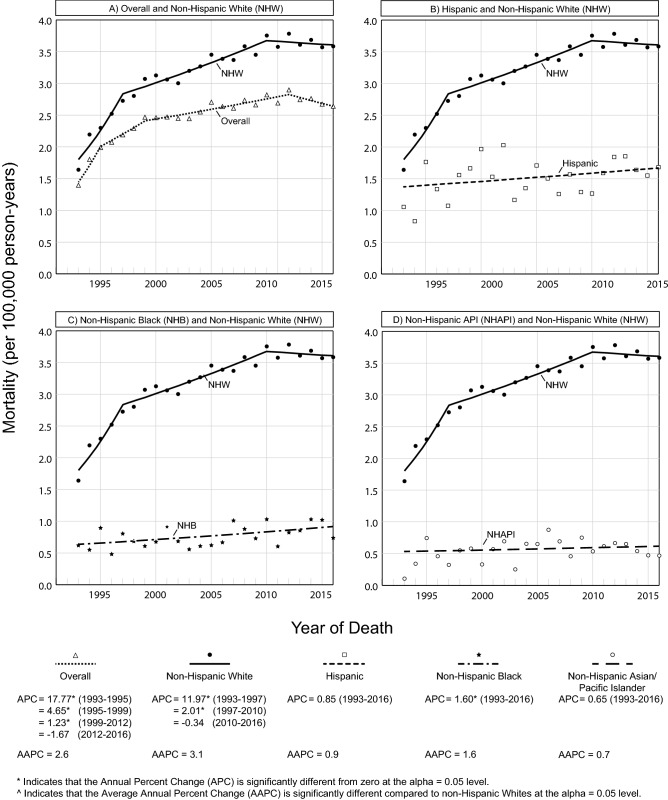
Racial and ethnic trends in EAC mortality, SEER 13 (1993–2016)

Figure 1 provides trends in mortality for EAC by race/ethnicity over the study period. Overall EAC mortality increased significantly from 1993 to 1995 (APC = 17.77, *p* = 0.003), from 1995 to 1999 (APC = 4.65, *p* = 0.03), and from 1999 to 2012 (APC = 1.23, *p* < 0.001) but stabilized from 2012 to 2016 (APC = − 1.67, *p* = 0.09). Similarly, NHW EAC mortality increased significantly from 1993 to 1997 (APC = 11.97, *p* < 0.001) and from 1997 to 2010 (APC = 2.01, *p* < 0.001) but stabilized from 2010 to 2016 (APC = − 0.34, *p* = 0.66). EAC mortality remained stable throughout the entire period for Hispanics (APC = 0.85, *p* = 0.13), NHAPI (APC = 0.65, *p* = 0.50), and NHAI/AN (APC = 2.26, *p* = 0.12) (Supplementary Table 1). Average trends in EAC mortality were not significantly different for each racial/ethnic group when compared to NHW (Fig. 1). At the end of the study period, only NHB had significantly increasing mortality (APC = 1.60, *p* = 0.01). Nonetheless, EAC mortality remained higher among NHW than among NHB at the end of the study period (NHW vs. NHB: 3.6 vs. 0.7 deaths per 100,000) (Supplementary Table 2).

### Trends in ESCC mortality overall and by race/ethnicity

**Fig. 2:**
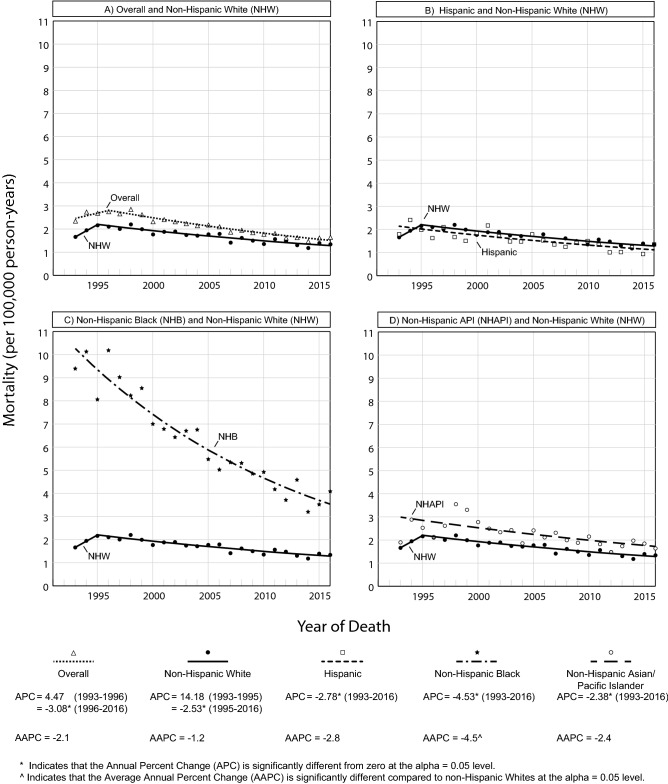
Racial and ethnic trends in ESCC mortality, SEER 13 (1993–2016)

Figure 2 demonstrates trends in ESCC mortality by race/ethnicity over the study period. Overall ESCC mortality was stable from 1993 to 1996 (APC = 4.47, *p* = 0.21) but decreased significantly from 1996 to 2016 (APC = − 3.08, *p* < 0.001). ESCC mortality decreased significantly throughout the entire period for Hispanics (APC = − 2.78 *p* < 0.001), NHB (APC = − 4.53, *p* < 0.001), and NHAPI (APC = − 2.38, *p* < 0.001). For NHW, ESCC mortality was stable from 1993 to 1995 (APC = 14.18, *p* = 0.17) and then declined significantly from 1995 to 2016 (APC = − 2.53, *p* < 0.001). For NHAI/AN, mortality remained stable (APC = − 1.79, *p* = 0.25) (Supplementary Table 1). NHB experienced the largest decrease in ESCC mortality (APC = − 4.53, *p* < 0.001), and this decreasing trend was significantly different compared to the average mortality trend in NHW (*p* < 0.001); however, ESCC mortality remained higher among NHB than among NHW at the end of the study period (NHB vs. NHW: 4.09 vs. 1.34 deaths per 100,000) (Supplementary Table 3).

### Trends in EAC stage at diagnosis overall and by race/ethnicity

**Fig. 3:**
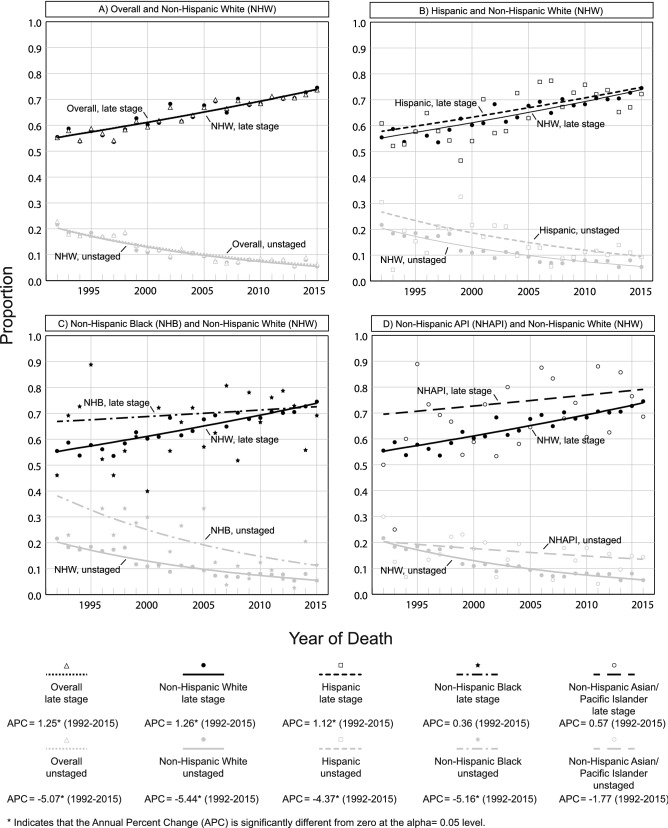
Racial and ethnic trends in EAC stage at diagnosis, SEER 13 (1992–2015)

Overall, the proportion of late stage EAC cases increased significantly throughout the entire study period (APC = 1.25, *p* < 0.001) (Fig. 3). In 1992, 55.2% of cases were late stage while 73.7% were late stage by 2015 (Supplementary Table 4). By race/ethnicity, there was a steady increase in late stage diagnoses in NHW (APC = 1.26, *p* < 0.001) and Hispanics (APC = 1.12, *p* = 0.002). Late stage proportions in NHAPI (APC = 0.57, *p* = 0.31) and NHB (APC = 0.36, *p* = 0.49) remained stable throughout the study period. EAC counts by stage were too small to perform trend analyses for NHAI/AN.

Of note, trends for unstaged EAC opposed trends in late stage EAC disease. The overall proportion of unstaged EAC decreased significantly throughout the study period (APC = − 5.07, *p* < 0.001), leading to a 16.5 percentage point decrease between 1992 and 2015 (Supplementary Table 4). This downward trend was significant among NHW (APC = − 5.44, *p* < 0.001), Hispanics (APC = − 4.37, *p* < 0.001), and NHB (APC = − 5.16, *p* = 0.001). Unstaged disease among NHAPI remained stable throughout the study period (APC = − 1.77, *p* = 0.11).

### Trends in ESCC stage at diagnosis overall and by race/ethnicity

**Fig. 4:**
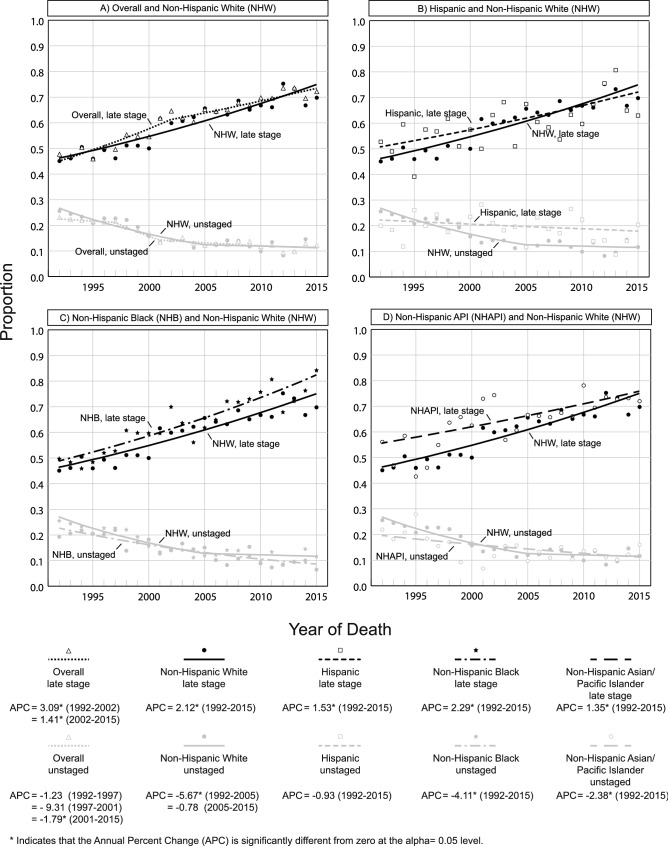
Racial and ethnic trends in ESCC stage at diagnosis, SEER 13 (1992–2015)

The proportion of late stage ESCC increased significantly from 1992 to 2002 (APC = 3.09, *p* < 0.001) and from 2002 to 2015 (APC = 1.41, *p* < 0.001) (Fig. 4). This trend amounted to an increase of 24.5% points between 1992 and 2015 (Supplementary Table 5). Late stage ESCC disease also increased significantly in NHW (APC = 2.12, *p* < 0.001), Hispanics (APC = 1.53, *p* < 0.001), NHB (APC = 2.29, *p* < 0.001), and NHAPI (APC = 1.35, *p* < 0.001). ESCC counts by stage were too small to perform trend analyses for NHAI/AN.

The overall proportion of unstaged disease remained statistically stable from 1992 to 1997 (APC = − 1.23, *p* = 0.50) and from 1997 to 2001 (APC = − 9.31, *p* = 0.06) before decreasing significantly from 2001 to 2015 (APC = − 1.79, *p* = 0.01). As a result, the proportion of individuals with unstaged disease decreased by 10.9% points between 1992 and 2015 (Supplementary Table 5). In NHW, the proportion of unstaged disease decreased significantly from 1992 to 2005 (APC = − 5.67, *p* < 0.001) before stabilizing from 2005 to 2015 (APC = − 0.78, *p* = 0.66). Unstaged disease decreased significantly throughout the entire period in NHB (APC = − 4.11, *p* < 0.001) and NHAPI (APC = − 2.38, *p* = 0.003), while remaining stable in Hispanics (APC = − 0.93, *p* = 0.22).

## Discussion

In summary, we found that all-cause mortality increased significantly among U.S. adults with EAC from 1993 to 2012 before stabilizing. In contrast, mortality decreased steadily from 1996 to 2016 among adults with ESCC. Increasing EAC mortality was driven by two racial/ethnic groups—NHW and NHB—while the recent stabilization appears to have been driven by stable trends in NHW in recent years. At the end of the study period, EAC mortality continued to increase significantly only for NHB. In contrast to EAC, the steep decline in ESCC mortality observed appears to have been driven by an impressive and steady decrease in mortality among NHB over the study period. Despite this decline in ESCC mortality among NHB, NHB maintained the highest mortality burden at the end of the study period. It is encouraging, however, that the disparity in ESCC mortality between NHB and NHW has narrowed.

Our analyses of stage at diagnosis revealed an upward trend in the proportion of late stage disease for both EAC and ESCC over the study period. Conversely, unstaged disease decreased over the study period for EAC and from 2001 to 2015 for ESCC. Trends in late stage and unstaged disease by race/ethnicity largely mirrored overall trends.

Changes in the prevalence of known EAC risk factors—obesity, GERD, poor diet, sedentary lifestyle, and use of medications that relax the lower esophageal sphincter—shape trends in incidence and mortality [3, 4]. Some have reported increased incidence of Barrett’s esophagus (BE) [23], supporting the well-established hypothesis that it is a major EAC risk factor [4]. ACG practice guidelines recommend screening for BE in men with chronic and/or frequent symptoms of GERD who have multiple risk factors [24]. Because White race is a known risk factor for development of BE, targeted screening recommendations intended to reduce the incidence of BE in NHW may disproportionately impact minorities. Future studies designed to understand why White race appears to be a strong risk factor for development BE are needed to further reduce this disparity. While these risk factors affect overall EAC incidence and mortality, there are likely additional contributors to the sustained rise in mortality observed in NHB. Disparities in mortality might be the result of differences in disease stage at diagnosis, management and treatment, or disease progression. Although NHB and NHW had similar proportions of late stage EAC, NHB appear to have higher rates of unstaged EAC than other groups, which might reflect a lower likelihood to pursue surgical treatment and higher treatment-related mortality [25, 26]. The degree to which these factors contribute to sustained increases in EAC mortality among NHB requires further investigation.

Decreasing mortality among individuals with ESCC coincides with decreasing ESCC incidence (Supplementary Table 1) [6]. A 2001 population-based study identified four major risk factors that accounted for 99% of the excess ESCC incidence observed in Black men: low income, moderate/heavy alcohol intake, tobacco use, and infrequent consumption of fruits and vegetables [27]. Heavy tobacco use has decreased among minorities [28]. Thus, the observed decrease in ESCC mortality is likely, at least in part, driven by decreasing ESCC incidence and risk factor prevalence, especially among minority racial/ethnic groups. It is encouraging that NHB, the racial/ethnic group with the highest ESCC mortality, continues to experience the steepest mortality decline.

Improvements in diagnostic imaging may explain rising proportions of late stage EAC and ESCC over time. For both EC subtypes, the overall proportion of late stage disease increased slightly more than the overall proportion of unstaged disease decreased. Taken together, these patterns support a stage migration phenomenon with transition from unstaged and localized stage disease to late stage disease among EC subtypes [29, 30]. Thus, our findings suggest that advances in diagnostic imaging have enhanced staging accuracy [31]. Increasing availability of advanced imaging technologies like positron emission tomography (PET) have likely resulted in more accurate characterization of disease and proportionally fewer cases of unstaged and localized stage disease over time [29, 30]. Inequities in factors that limit access to health care and utilization, such as adequate health insurance, comorbidity, care-seeking behavior, socioeconomic status, and education status, may also contribute to relative differences in stage at diagnosis by race/ethnicity [32–34]. Future studies should further investigate the degree to which these factors contribute to disparities in relative disease staging.

This study has several strengths. First, this is the first study to use a large and diverse U.S. cohort to describe trends in mortality and stage at diagnosis for individuals with EC. Second, consistent with strong evidence that EAC and ESCC have different risk factors, genetic factors, and epidemiology, we stratified our analyses by histological subtype. Third, the SEER database provides access to high-quality cancer registry data with national representation and the ability to evaluate cancer indicators over time [35]. Lastly, we evaluated our outcomes of interest by race/ethnicity using the earliest SEER data available for Hispanics, NHAPI, and NHAI/AN, which contributes new knowledge to the literature about disparities in EAC and ESCC.

Our study is not without limitations. SEER does not capture data by nationality, immigration status, or immigrant generation, which are helpful to fully understand the impact of geographic origin and acculturation on disease outcomes. Further, SEER uses the North American Association of Central Cancer Registries (NACCR) Hispanic Identification Algorithm, which uses indirect variables to classify persons as Hispanic/Latino or non-Hispanic when direct variables on Hispanic/Latino are not available [36]. This potentially results in misclassification of Hispanic ethnicity. We also acknowledge that data on cause-specific mortality are helpful to isolate mortality trends related to esophagectomy, local excision, chemoradiation, and other primary treatment modalities, which might help further understand racial/ethnic disparities in EC mortality. This study was also limited by sparse counts of EC subtypes in NHAI/AN. Lastly, we note that SEER 13 only represents 13.4% of the U.S population; thus, our findings might not be generalizable to every region of the country. Nonetheless, SEER is one of few data sources available to investigate national cancer trends and disparities.

In conclusion, our study contributes to the literature a summary of recent U.S trends in EC mortality and stage at diagnosis by major histological subtype and race/ethnicity. There are notable persistent racial/ethnic disparities in both EAC and ESCC mortality, with the highest mortality among NHW individuals with EAC and NHB individuals with ESCC. However, it is promising that EAC mortality has stabilized in almost every racial/ethnic group and that ESCC mortality is decreasing in all racial/ethnic groups. Further, differences in ESCC mortality between NHB and NHW and between Hispanics and NHW are narrowing. Future efforts should aim to identify specific genetic, environmental, and healthcare related factors that drive current disparities. In addition, there should be a specific focus on reducing mortality among NHB with EAC, for whom mortality is rising. Lastly, our findings provide a strong basis for future studies to investigate contributors to stage migration and racial/ethnic variation in stage at EC diagnosis. To improve EAC and ESCC outcomes nationally, we must identify and address modifiable risk factors to prevent EC, develop strategies to increase early EC detection, and ensure equitable access to diagnostic testing and treatment options for all racial/ethnic groups.

## Supplementary Information

Below is the link to the electronic supplementary material.

Supplementary file1 (PDF 300 KB)

## Data Availability

Available upon request.
