# Supplementary material for: Trends in Esophageal Cancer Mortality and Stage at Diagnosis by Race and Ethnicity in the United States
Source: Cancer Causes Control. 2021 May 18;32(8):883–94. doi: 10.1007/s10552-021-01443-z (PMC8236464; doi:10.1007/s10552-021-01443-z)
Supplement: Supplementary file 1 — Supplementary file1 (PDF 300 KB) [file 10552_2021_1443_MOESM1_ESM.pdf]

Supplementary Table 1: Trends in EAC and ESCC incidence and mortality by race/ethnicity in the United States

	Trend 1		Trend 2		Trend 3		Trend 4		Average APC
	Years	APC	Years	APC	Years	APC	Years	APC	Entire study period¥
**EAC**									
**Incidence**									
Overall	1992-2004	3.15*	2004-2016	0.00					1.6*
NHW	1992-1999	6.14*	1999-2016	0.97*					2.4*
Hispanic	1992-2016	1.04							1.0
NHB	1992-2016	1.80*							1.8*
NHAPI	1992-2016	1.62*							1.6*
NHAI/AN	1992-2016	2.36*							2.4*
**Mortality**									
Overall	1993-1995	17.77*	1995-1999	4.65*	1999-2012	1.23*	2012-2016	-1.67	2.6*
NHW	1993-1997	11.97*	1997-2010	2.01*	2010-2016	-0.34			3.1*
Hispanic	1993-2016	0.85							0.9*
NHB	1993-2016	1.60*							1.6*
NHAPI	1993-2016	0.65							0.7
NHAI/AN	1993-2016	2.26							2.3
**ESCC**									
**Incidence**									
Overall	1992-2016	-3.18*							-3.2*
NHW	1992-2016	-2.57*							-2.6*
Hispanic	1992-2016	-3.11*							-3.1*
NHB	1992-2016	-5.27*							-5.3*
NHAPI	1992-2016	-2.57*							-2.6*
NHAI/AN	1992-2016	-1.60							-1.6
**Mortality**									
Overall IBM	1993-1996	4.47	1996-2016	-3.08*					-2.1*
NHW IBM	1993-1995	14.18	1995-2016	-2.53*					-1.2
Hispanic IBM	1993-2016	-2.78*							-2.8*
NHB IBM	1993-2016	-4.53*							-4.5*
NHAPI IBM	1993-2016	-2.38*							-2.4*
NHAI/AN IBM	1993-2016	-1.79							-1.8

Average incidence rates are age-adjusted to the 2000 US standard population.

APC, annual percent change; EAC, esophageal adenocarcinoma; ESCC, esophageal squamous cell carcinoma; IR, incidence rate; NHW, non-Hispanic White; NHB, non-Hispanic Black; NHAPI, non-Hispanic Asian/Pacific Islander; NHAI/AN, non-Hispanic American Indian/Alaskan Native.

*The APC is significantly different from 0.0 (P < .05).

¥The entire study period for incidence rate is from 1992-2016 while the entire study period for incidence-based mortality is from 1993-2016.

Supplementary Table 2: Esophageal adenocarcinoma annual mortality rates (95% Cis) in the United States by race/ethnicity

Year of Death	Overall	NHW	Hispanic	NHB	NHAPI	NHAI/AN
1993-2016	2.5 (2.5-2.5)	3.2 (3.1-3.2)	1.5 (1.4-1.6)	0.8 (0.7-0.9)	0.6 (0.5-0.6)	1.8 (1.4-2.2)
1993	1.4 (1.24-1.56)	1.64 (1.45-1.85)	1.06 (0.61-1.68)	0.62 (0.32-1.09)	0.11 (0.01-0.38)	0.8 (0.02-4.05)
1994	1.8 (1.63-1.99)	2.2 (1.98-2.43)	0.83 (0.49-1.32)	0.55 (0.27-1)	0.34 (0.14-0.71)	1.79 (0.35-5.34)
1995	1.99 (1.81-2.18)	2.3 (2.08-2.54)	1.76 (1.18-2.51)	0.9 (0.51-1.45)	0.74 (0.4-1.26)	1.73 (0.2-5.68)
1996	2.07 (1.89-2.27)	2.52 (2.29-2.77)	1.34 (0.87-1.95)	0.48 (0.22-0.92)	0.46 (0.22-0.84)	1.74 (0.2-5.68)
1997	2.19 (2-2.39)	2.73 (2.49-2.98)	1.07 (0.64-1.67)	0.81 (0.46-1.31)	0.32 (0.13-0.67)	0.49 (0.01-2.94)
1998	2.29 (2.1-2.49)	2.8 (2.56-3.06)	1.56 (1.04-2.22)	0.69 (0.37-1.16)	0.55 (0.28-0.96)	1.41 (0.16-4.79)
1999	2.47 (2.27-2.67)	3.07 (2.82-3.34)	1.66 (1.14-2.33)	0.61 (0.31-1.06)	0.58 (0.31-0.97)	0.44 (0.01-2.72)
2000	2.46 (2.27-2.66)	3.13 (2.87-3.4)	1.97 (1.37-2.71)	0.68 (0.37-1.14)	0.33 (0.14-0.65)	0.39 (0.01-2.51)
2001	2.47 (2.28-2.67)	3.06 (2.81-3.33)	1.53 (1.05-2.14)	0.91 (0.55-1.42)	0.57 (0.32-0.94)	2.08 (0.51-5.42)
2002	2.45 (2.26-2.65)	3 (2.76-3.27)	2.03 (1.49-2.69)	0.69 (0.38-1.14)	0.69 (0.41-1.08)	1.66 (0.2-5.25)
2003	2.45 (2.26-2.64)	3.2 (2.94-3.47)	1.17 (0.78-1.67)	0.56 (0.29-0.98)	0.25 (0.1-0.52)	2.34 (0.58-5.93)
2004	2.55 (2.36-2.75)	3.27 (3.01-3.54)	1.35 (0.95-1.86)	0.61 (0.33-1.03)	0.65 (0.39-1.02)	1.51 (0.28-4.42)
2005	2.7 (2.51-2.91)	3.45 (3.19-3.73)	1.71 (1.24-2.28)	0.62 (0.34-1.04)	0.65 (0.39-1.01)	1.95 (0.61-4.7)
2006	2.64 (2.45-2.84)	3.39 (3.13-3.66)	1.5 (1.08-2.03)	0.67 (0.37-1.11)	0.88 (0.57-1.27)	2.32 (0.67-5.55)
2007	2.61 (2.42-2.81)	3.37 (3.11-3.65)	1.26 (0.89-1.72)	1.01 (0.64-1.52)	0.69 (0.44-1.04)	0.98 (0.2-3.11)
2008	2.74 (2.55-2.94)	3.59 (3.32-3.87)	1.57 (1.15-2.08)	0.88 (0.54-1.35)	0.46 (0.26-0.74)	1.48 (0.39-3.94)
2009	2.66 (2.48-2.86)	3.45 (3.19-3.73)	1.29 (0.95-1.71)	0.73 (0.43-1.17)	0.75 (0.49-1.1)	3.94 (1.77-7.47)
2010	2.82 (2.63-3.02)	3.75 (3.49-4.04)	1.27 (0.92-1.69)	1.03 (0.68-1.5)	0.54 (0.33-0.83)	2.9 (0.99-6.29)
2011	2.69 (2.51-2.89)	3.58 (3.32-3.85)	1.59 (1.21-2.04)	0.61 (0.36-0.97)	0.62 (0.39-0.92)	1.33 (0.43-3.35)
2012	2.9 (2.71-3.1)	3.78 (3.52-4.06)	1.84 (1.43-2.33)	0.83 (0.52-1.25)	0.67 (0.44-0.97)	1.91 (0.56-4.58)
2013	2.74 (2.56-2.93)	3.61 (3.35-3.88)	1.85 (1.44-2.34)	0.86 (0.54-1.28)	0.65 (0.43-0.95)	1.62 (0.48-3.95)
2014	2.76 (2.58-2.95)	3.69 (3.43-3.96)	1.64 (1.27-2.08)	1.03 (0.69-1.49)	0.54 (0.34-0.81)	2.09 (0.71-4.67)
2015	2.67 (2.5-2.86)	3.57 (3.32-3.84)	1.55 (1.19-1.97)	1.02 (0.7-1.45)	0.47 (0.3-0.71)	2.32 (0.9-4.85)
2016	2.64 (2.47-2.82)	3.58 (3.34-3.85)	1.68 (1.31-2.12)	0.74 (0.46-1.13)	0.47 (0.29-0.71)	1.79 (0.61-4.04)

95% CI, 95% confidence interval; NHW, Non-Hispanic White; NHB, Non-Hispanic Black; NHAPI, Non-Hispanic Asian and Pacific Islander; NHAI/AN, Non-Hispanic American Indian/Alaska Native.
Rates are age-adjusted to the 2000 US standard population (19 age groups-census p25-1130) standard.

Supplementary Table 3: Esophageal squamous cell carcinoma age-adjusted annual mortality rates (95% CIs) in the United States by race/ethnicity

Year of Death	Overall	NHW	Hispanic	NHB	NHAPI	NHAI/AN
**1993-2016**	2.1 (2.1-2.1)	1.7 (1.6-1.7)	1.5 (1.4-1.6)	5.9 (5.7-6.1)	2.2 (2.1-2.3)	2.5 (2.1-3.0)
**1993**	2.34 (2.15-2.55)	1.66 (1.47-1.87)	1.79 (1.2-2.55)	9.4 (8.04-10.92)	1.9 (1.32-2.64)	2.75 (0.69-7.08)
**1994**	2.74 (2.52-2.96)	1.95 (1.74-2.17)	2.41 (1.74-3.24)	10.14 (8.72-11.71)	2.88 (2.13-3.81)	2.03 (0.36-5.99)
**1995**	2.68 (2.47-2.9)	2.16 (1.95-2.39)	1.99 (1.36-2.78)	8.07 (6.84-9.45)	2.53 (1.85-3.37)	2.12 (0.57-5.57)
**1996**	2.75 (2.54-2.97)	2.1 (1.89-2.33)	1.63 (1.1-2.31)	10.19 (8.8-11.73)	2.13 (1.54-2.87)	1.3 (0.15-4.54)
**1997**	2.66 (2.45-2.87)	2.01 (1.8-2.23)	2.1 (1.49-2.86)	9.03 (7.74-10.47)	2.62 (1.95-3.42)	2.1 (0.4-5.97)
**1998**	2.85 (2.64-3.07)	2.2 (1.99-2.43)	1.67 (1.15-2.34)	8.24 (7.03-9.59)	3.55 (2.8-4.43)	7.36 (3.6-13.07)
**1999**	2.62 (2.42-2.83)	1.99 (1.79-2.21)	1.51 (1.01-2.15)	8.56 (7.31-9.95)	3.31 (2.59-4.14)	2.19 (0.57-5.61)
**2000**	2.32 (2.13-2.52)	1.77 (1.58-1.97)	1.85 (1.31-2.52)	7.01 (5.89-8.27)	2.78 (2.15-3.52)	1.78 (0.34-5.1)
**2001**	2.41 (2.22-2.61)	1.88 (1.68-2.09)	2.17 (1.6-2.87)	6.79 (5.72-8.01)	2.49 (1.92-3.17)	2.4 (0.76-5.64)
**2002**	2.32 (2.13-2.51)	1.89 (1.7-2.11)	1.71 (1.21-2.34)	6.44 (5.4-7.61)	2.34 (1.79-3)	4.88 (1.88-9.84)
**2003**	2.23 (2.06-2.42)	1.74 (1.56-1.95)	1.48 (1.04-2.03)	6.71 (5.66-7.9)	2.43 (1.88-3.08)	3.43 (1.29-7.19)
**2004**	2.15 (1.97-2.33)	1.71 (1.53-1.92)	1.48 (1.02-2.05)	6.76 (5.71-7.94)	1.86 (1.39-2.42)	3.11 (1.04-6.88)
**2005**	2.18 (2-2.36)	1.77 (1.58-1.97)	1.79 (1.31-2.37)	5.48 (4.55-6.55)	2.41 (1.89-3.04)	2.58 (0.92-5.69)
**2006**	2.1 (1.93-2.28)	1.79 (1.6-2)	1.53 (1.09-2.06)	5.03 (4.14-6.05)	2.11 (1.63-2.69)	4.4 (2-8.28)
**2007**	1.87 (1.71-2.03)	1.41 (1.25-1.59)	1.35 (0.96-1.83)	5.35 (4.45-6.38)	2.31 (1.81-2.91)	2.13 (0.52-5.42)
**2008**	1.95 (1.79-2.12)	1.62 (1.44-1.81)	1.25 (0.87-1.73)	5.32 (4.42-6.33)	1.99 (1.54-2.53)	2.86 (0.97-6.3)
**2009**	1.85 (1.69-2.01)	1.5 (1.33-1.68)	1.42 (1.03-1.9)	4.86 (4.02-5.82)	1.88 (1.45-2.39)	1.95 (0.48-4.96)
**2010**	1.77 (1.62-1.94)	1.35 (1.19-1.53)	1.5 (1.1-1.98)	4.93 (4.08-5.89)	2.15 (1.7-2.68)	1.05 (0.13-3.51)
**2011**	1.81 (1.66-1.97)	1.56 (1.39-1.75)	1.34 (0.97-1.79)	4.18 (3.43-5.05)	1.82 (1.42-2.3)	1.63 (0.47-4.04)
**2012**	1.62 (1.48-1.77)	1.48 (1.31-1.66)	1.01 (0.7-1.4)	3.72 (3.01-4.55)	1.47 (1.11-1.9)	1.45 (0.36-3.83)
**2013**	1.63 (1.49-1.78)	1.3 (1.15-1.47)	1.02 (0.71-1.4)	4.59 (3.82-5.47)	1.73 (1.35-2.18)	3.03 (1.23-6.08)
**2014**	1.47 (1.34-1.61)	1.18 (1.04-1.34)	1.23 (0.91-1.62)	3.21 (2.58-3.93)	1.97 (1.58-2.44)	0.26 (0.01-1.73)
**2015**	1.61 (1.48-1.76)	1.39 (1.24-1.56)	0.94 (0.67-1.28)	3.53 (2.86-4.3)	1.85 (1.47-2.29)	3.32 (1.45-6.38)
**2016**	1.64 (1.51-1.78)	1.34 (1.19-1.51)	1.35 (1.02-1.76)	4.09 (3.4-4.89)	1.64 (1.29-2.05)	2.2 (0.91-4.51)

95% CI, 95% confidence interval; NHW, Non-Hispanic White; NHB, Non-Hispanic Black; NHAPI, Non-Hispanic Asian and Pacific Islander; NHAI/AN, Non-Hispanic American Indian/Alaska Native.
Rates are age-adjusted to the 2000 US standard population (19 age groups-census p25-1130) standard.

Supplementary Table 4: Esophageal adenocarcinoma annual proportion of unstaged and late stage disease at diagnosis in the United States by race/ethnicity

	Overall		NHW		Hispanic		NHB		NHAPI	
**Year of diagnosis**	**Proportion Unstaged (SE)**	**Proportion Late Stage (SE)**	**Proportion Unstaged (SE)**	**Proportion Late Stage (SE)**	**Proportion Unstaged (SE)**	**Proportion Late Stage (SE)**	**Proportion Unstaged (SE)**	**Proportion Late Stage (SE)**	**Proportion Unstaged (SE)**	**Proportion Late Stage (SE)**
**1992-2015**	10.8 (0.2)	65.5 (0.3)	10.4 (0.2)	65.3 (0.4)	13.6 (1.0)	66.6 (1.3)	15.6 (1.6)	65.3 (2.1)	12.6 (1.5)	69.2 (2.1)
**1992**	22.9 (1.9)	55.2 (2.3)	21.7 (2)	55.5 (2.4)	30.4 (9.6)	60.9 (10.2)	46.2 (13.8)	46.2 (13.8)	30 (14.5)	50 (15.8)
**1993**	17.9 (1.7)	57.9 (2.2)	18.3 (1.9)	58.7 (2.4)	4.3 (4.3)	52.2 (10.4)	23.1 (11.7)	69.2 (12.8)	12.5 (11.7)	25 (15.3)
**1994**	17.3 (1.7)	54.1 (2.2)	17.4 (1.8)	53.7 (2.4)	19.4 (6.6)	52.8 (8.3)	18.2 (11.6)	72.7 (13.4)	6.7 (6.4)	60 (12.6)
**1995**	17.5 (1.7)	58.8 (2.2)	18.5 (1.8)	57.7 (2.3)	15.4 (7.1)	57.7 (9.7)	0 (0)	88.9 (10.5)	0 (0)	88.9 (10.5)
**1996**	17 (1.5)	56.8 (1.9)	16.8 (1.6)	56.1 (2.1)	10.8 (5.1)	64.9 (7.8)	33.3 (10.3)	52.4 (10.9)	13.3 (8.8)	73.3 (11.4)
**1997**	17.6 (1.5)	54.1 (2)	17.4 (1.6)	53.6 (2.1)	21.1 (6.6)	57.9 (8)	23.1 (11.7)	46.2 (13.8)	15.4 (10)	69.2 (12.8)
**1998**	18.7 (1.5)	58.3 (1.9)	18.2 (1.6)	58.4 (2)	20 (6.8)	54.3 (8.4)	33.3 (15.7)	55.6 (16.6)	22.2 (13.9)	66.7 (15.7)
**1999**	13.5 (1.3)	61.6 (1.8)	11.7 (1.3)	62.7 (1.9)	32.6 (7.1)	46.5 (7.6)	27.8 (10.6)	61.1 (11.5)	23.1 (11.7)	53.8 (13.8)
**2000**	12.1 (1.2)	59.3 (1.8)	10.9 (1.2)	60.2 (1.9)	21.6 (6.8)	54.1 (8.2)	30 (10.2)	40 (11)	17.6 (9.2)	58.8 (11.9)
**2001**	12 (1.2)	61.8 (1.8)	11.6 (1.3)	61 (1.9)	17 (5.5)	70.2 (6.7)	11.1 (7.4)	72.2 (10.6)	20 (10.3)	73.3 (11.4)
**2002**	9.7 (1.1)	66.9 (1.7)	8.8 (1.1)	68.3 (1.8)	21.4 (6.3)	57.1 (7.6)	16.7 (8.8)	55.6 (11.7)	6.7 (6.4)	53.3 (12.9)
**2003**	12.2 (1.2)	61.7 (1.8)	11.2 (1.2)	61.5 (1.9)	21.1 (6.6)	57.9 (8)	26.7 (11.4)	66.7 (12.2)	13.3 (8.8)	80 (10.3)
**2004**	10.7 (1)	63.9 (1.6)	10.8 (1.1)	63.2 (1.7)	9.7 (3.8)	72.6 (5.7)	0 (0)	72.2 (10.6)	19.4 (7.1)	58.1 (8.9)
**2005**	9.9 (1)	66.9 (1.6)	9.4 (1.1)	67.7 (1.7)	12.9 (4.3)	62.9 (6.1)	33.3 (10.3)	57.1 (10.8)	0 (0)	64.5 (8.6)
**2006**	7.5 (0.9)	69.9 (1.6)	7.3 (1)	69.2 (1.7)	12.8 (5.4)	76.9 (6.7)	12.5 (8.3)	62.5 (12.1)	0 (0)	87.5 (6.8)
**2007**	6.8 (0.8)	66.5 (1.6)	7 (0.9)	64.9 (1.7)	5.7 (3.2)	77.4 (5.7)	3.8 (3.8)	80.8 (7.7)	5.6 (5.4)	83.3 (8.8)
**2008**	7.4 (0.8)	69.5 (1.5)	6.8 (0.9)	70.3 (1.6)	8.6 (3.7)	67.2 (6.2)	11.1 (6)	51.9 (9.6)	17.9 (7.2)	67.9 (8.8)
**2009**	8.2 (0.9)	68.4 (1.5)	8 (0.9)	67.8 (1.6)	9.1 (3.3)	72.7 (5.1)	6.3 (4.3)	78.1 (7.3)	13 (7)	73.9 (9.2)
**2010**	8.6 (0.9)	68.4 (1.5)	8 (1)	68.3 (1.6)	11.3 (4)	75.8 (5.4)	12.5 (6.8)	66.7 (9.6)	17.9 (7.2)	60.7 (9.2)
**2011**	8 (0.8)	71.2 (1.4)	7.7 (0.9)	70.6 (1.5)	11.6 (3.5)	72.1 (4.8)	9.5 (6.4)	76.2 (9.3)	4 (3.9)	88 (6.5)
**2012**	8.1 (0.8)	70.7 (1.4)	7.8 (0.9)	70.2 (1.5)	10.1 (3)	73.7 (4.4)	6.1 (4.2)	78.8 (7.1)	15.6 (6.4)	62.5 (8.6)
**2013**	5.9 (0.7)	70.6 (1.4)	5.4 (0.7)	70.5 (1.5)	13.9 (4.1)	65.3 (5.6)	2.7 (2.7)	73 (7.3)	9.5 (6.4)	85.7 (7.6)
**2014**	8.9 (0.9)	71.8 (1.4)	8 (0.9)	72.7 (1.5)	11 (3.5)	67.1 (5.2)	20.6 (6.9)	55.9 (8.5)	14.7 (6.1)	76.5 (7.3)
**2015**	6.4 (0.7)	73.7 (1.3)	5.4 (0.7)	74.5 (1.4)	9.3 (2.8)	72.2 (4.3)	11.5 (6.3)	69.2 (9.1)	14.3 (5.9)	68.6 (7.8)

NHW, Non-Hispanic White; NHB, Non-Hispanic Black; NHAPI, Non-Hispanic Asian and Pacific Islander.
SE, standard error
Counts by year were to sparse for Non-Hispanic American Indian/Alaska Native.

Supplementary Table 5: Esophageal squamous cell carcinoma annual proportion of late and unstaged disease at diagnosis in the United States by race/ethnicity

	Overall		NHW		Hispanic		NHB		NHAPI	
Year of diagnosis	Proportion Unstaged (SE)	Proportion Late Stage (SE)	Proportion Unstaged (SE)	Proportion Late Stage (SE)	Proportion Unstaged (SE)	Proportion Late Stage (SE)	Proportion Unstaged (SE)	Proportion Late Stage (SE)	Proportion Unstaged (SE)	Proportion Late Stage (SE)
1992-2015	16.1 (0.3)	60.1 (0.4)	16.4 (0.4)	58.4 (0.5)	19.2 (1.2)	59.7 (1.5)	15.2 (0.6)	61.5 (0.8)	14.3 (0.8)	64.8 (1.1)
1992	23.1 (1.5)	47.7 (1.8)	25.6 (2.1)	45.1 (2.4)	20 (5.4)	52.7 (6.7)	19.3 (2.6)	49.8 (3.3)	22 (6.5)	56.1 (7.8)
1993	22.3 (1.5)	47 (1.8)	24.5 (2.1)	46.1 (2.4)	18.4 (5.5)	49 (7.1)	20.5 (2.7)	48.4 (3.4)	18.2 (4.7)	47 (6.1)
1994	22 (1.6)	50.4 (1.9)	23.4 (2.1)	50.5 (2.5)	11.9 (5)	59.5 (7.6)	21.7 (2.9)	46 (3.5)	20.8 (5.6)	58.5 (6.8)
1995	21.7 (1.5)	45.9 (1.8)	20.8 (2)	46 (2.4)	26.1 (6.5)	39.1 (7.2)	20.4 (2.9)	48.5 (3.6)	27.9 (5.7)	42.6 (6.3)
1996	21.3 (1.5)	50.6 (1.9)	22.7 (2.1)	49.4 (2.5)	20 (6.3)	57.5 (7.8)	20.3 (2.7)	52.1 (3.4)	18.4 (4.4)	46.1 (5.7)
1997	21.7 (1.6)	49.6 (1.9)	22.7 (2.1)	46.1 (2.5)	24.3 (7.1)	56.8 (8.1)	21.9 (3.1)	52.8 (3.7)	15.5 (4.3)	54.9 (5.9)
1998	19.2 (1.5)	55.3 (1.9)	22.1 (2.1)	51.2 (2.5)	17.6 (6.5)	61.8 (8.3)	13.9 (2.7)	60.8 (3.8)	17 (4)	63.6 (5.1)
1999	17.9 (1.5)	54.7 (1.9)	19.4 (2)	51.1 (2.6)	20.8 (5.6)	50.9 (6.9)	17.1 (3.1)	59.9 (4)	9.2 (3.3)	65.8 (5.4)
2000	17.1 (1.5)	54.6 (2)	15.8 (2)	50 (2.7)	23.4 (6.2)	57.4 (7.2)	18.2 (3.1)	59.7 (4)	16.7 (4.4)	62.5 (5.7)
2001	13.4 (1.3)	61.8 (1.9)	13.4 (1.7)	61.6 (2.5)	28.3 (6.6)	50 (7.4)	12.8 (2.7)	59.7 (4)	6.8 (2.9)	73 (5.2)
2002	14.4 (1.4)	64.6 (1.9)	14.3 (1.9)	59.9 (2.7)	21.1 (6.6)	63.2 (7.8)	14 (2.8)	70 (3.7)	11.5 (3.6)	74.4 (4.9)
2003	15.2 (1.5)	62 (2)	14.2 (1.9)	60.7 (2.7)	18.2 (5.8)	68.2 (7)	16.7 (2.9)	63.7 (3.7)	15.5 (4.8)	56.9 (6.5)
2004	13 (1.3)	60.3 (1.9)	11.3 (1.6)	62.2 (2.5)	19.6 (5.6)	51 (7)	14.4 (2.9)	56.2 (4.1)	15.7 (3.9)	60.7 (5.2)
2005	12.2 (1.4)	65.2 (2)	12 (1.8)	65.6 (2.7)	11.6 (4.9)	67.4 (7.1)	15.1 (3.2)	61.9 (4.3)	9.7 (3.5)	66.7 (5.6)
2006	12.8 (1.4)	64.5 (2)	12.3 (1.8)	64.2 (2.7)	23.3 (6.4)	60.5 (7.5)	10.2 (2.6)	65 (4.1)	13.3 (3.7)	66.3 (5.2)
2007	12.7 (1.4)	65.1 (2)	14.2 (2)	63.2 (2.7)	18.8 (5.6)	58.3 (7.1)	8.3 (2.4)	72.2 (3.9)	11 (3.7)	65.8 (5.6)
2008	13.9 (1.5)	67.8 (2)	14 (2)	68.6 (2.7)	17.1 (5.9)	53.7 (7.8)	10.7 (2.8)	71.9 (4.1)	15.1 (3.9)	67.4 (5.1)
2009	13.7 (1.4)	66.7 (1.9)	11.7 (1.7)	65.1 (2.6)	26.5 (6.3)	63.3 (6.9)	15.4 (3.2)	73.1 (3.9)	10.3 (3.7)	66.2 (5.7)
2010	11.7 (1.3)	69.7 (1.9)	9.9 (1.7)	66.8 (2.7)	22.4 (5.1)	59.7 (6)	8.9 (2.6)	75.8 (3.8)	13.5 (3.5)	78.1 (4.2)
2011	12.3 (1.4)	69.5 (2)	13.5 (1.9)	66.1 (2.6)	14 (5.3)	67.4 (7.1)	7.3 (2.5)	80.7 (3.8)	13.9 (4.1)	69.4 (5.4)
2012	9.4 (1.3)	73.5 (1.9)	8.3 (1.6)	75.2 (2.5)	14.3 (5)	75.5 (6.1)	8.5 (2.7)	67.9 (4.5)	11.1 (3.7)	73.6 (5.2)
2013	9.7 (1.2)	73.6 (1.8)	9.6 (1.7)	73.2 (2.5)	8.8 (3.7)	80.7 (5.2)	9.5 (2.6)	72.2 (4)	10.2 (3.2)	72.7 (4.7)
2014	13 (1.4)	69.5 (2)	14.6 (2.1)	66.8 (2.7)	14 (4.6)	64.9 (6.3)	10 (2.9)	76.4 (4.1)	12.2 (3.6)	73.2 (4.9)
2015	12.2 (1.3)	72.2 (1.8)	11.7 (1.8)	69.8 (2.5)	20.4 (5.5)	63 (6.6)	6.5 (2.4)	84.3 (3.5)	16 (3.7)	72 (4.5)

NHW, Non-Hispanic White; NHB, Non-Hispanic Black; NHAPI, Non-Hispanic Asian and Pacific Islander; NHAI/AN, Non-Hispanic American Indian/Alaska Native.
SE, standard error
Counts by year were to sparse for Non-Hispanic American Indian/Alaska Native.
